# Nanoparticles for Antimicrobial Agents Delivery—An Up-to-Date Review

**DOI:** 10.3390/ijms232213862

**Published:** 2022-11-10

**Authors:** Doina-Antonia Mercan, Adelina-Gabriela Niculescu, Alexandru Mihai Grumezescu

**Affiliations:** 1 Department of Science and Engineering of Oxide Materials and Nanomaterials, Politehnica University of Bucharest, 011061 Bucharest, Romania; 2 Research Institute of the University of Bucharest—ICUB, University of Bucharest, 050657 Bucharest, Romania; 3 Academy of Romanian Scientists, Ilfov No. 3, 050044 Bucharest, Romania

**Keywords:** antimicrobial therapy, antimicrobial resistance, drug delivery systems, antimicrobials delivery, nanocarriers, antibacterial nanoformulations, antifungal nanoformulations, antiviral nanoformulations, antiparasitic nanoformulations

## Abstract

Infectious diseases constitute an increasing threat to public health and medical systems worldwide. Particularly, the emergence of multidrug-resistant pathogens has left the pharmaceutical arsenal unarmed to fight against such severe microbial infections. Thus, the context has called for a paradigm shift in managing bacterial, fungal, viral, and parasitic infections, leading to the collision of medicine with nanotechnology. As a result, renewed research interest has been noted in utilizing various nanoparticles as drug delivery vehicles, aiming to overcome the limitations of current treatment options. In more detail, numerous studies have loaded natural and synthetic antimicrobial agents into different inorganic, lipid, and polymeric-based nanomaterials and tested them against clinically relevant pathogens. In this respect, this paper reviews the most recently reported successfully fabricated nanoformulations that demonstrated a great potential against bacteria, fungi, viruses, and parasites of interest for human medicine.

## 1. Introduction

Even though humans and pathogens have always dynamically interacted, this relationship became unbalanced. Human activities have caused pathogenic microbes, such as bacteria, fungi, viruses, and parasites, to appear and spread at a progressively distressing rate, rendering infectious diseases a common and burdensome health issue worldwide [1,2,3,4]. Numerous antimicrobial agents can be employed to fight against infections, yet they often face several limitations [5,6,7]. Specifically, the efficacy of conventional antimicrobial drugs is affected by their poor oral bioavailability and stability, low water solubility, low transportation rate across cellular membranes, lack of targeting, and systemic adverse effects [8,9,10,11]. Another important drawback of traditional drug-based therapeutic strategies is the inappropriate and inadequate administration of antimicrobial agents that have contributed to the emergence of drug-resistant pathogens and the formation of well-organized microbial communities called biofilms [12,13,14,15,16].

Unfortunately, biofilms have become a highly frequent problem in the clinical environment as microorganisms may adhere to and colonize the surfaces of biomedical devices. Thus, in order to avoid the acquiring of nosocomial infections, the burden of biofilms must be especially considered when using implantable and indwelling medical devices (e.g., catheters, stents, heart valves, pacemakers, prosthetic joints and implants, voice prostheses, contact lenses, internal and external fixation devices) [14,16,17,18,19,20,21,22,23]. Generally, the management of device-associated infections involves prolonged inpatient stay, surgical intervention, and long-term postoperative antibiotic therapy, all adding to healthcare costs and low patient compliance [10,13,24]. 

Despite existing therapies and medicines, infectious diseases in general, and biofilms in particular, remain difficult-to-eradicate problems [3]. Therefore, research efforts must be put into developing antimicrobial strategies able to surpass current challenges. In this context, the advances in nanotechnology represent promising opportunities for designing novel antimicrobial systems. A variety of nanoparticles (NPs) can be employed in developing performant delivery vehicles for natural and synthetic medicines capable of enhancing the activity of carried freight, ensuring a sustained drug release, and offering a chance of biofilm penetration and internalization into pathogenic microorganisms [8,25,26]. NPs loaded with antimicrobial agents can be used as effective therapeutics administered on different routes, but they can also be incorporated further into biomaterials for modifying surface nanotopography or coating biomedical devices, intending to potentiate or induce anti-infective properties [9,11,27,28,29,30,31]. 

In this respect, the present paper aims to overview the most recently developed antimicrobial nanoformulations, fabricated mainly between 2018 and 2022, that showed promising results when tested against clinically relevant pathogens, emphasizing their utility and versatility. Even though the topic has been previously addressed by several works [32,33,34,35,36], this review proposes a comprehensive path, correlating materials, fabrication methods, and delivered antimicrobial agents with targeted microorganisms, focusing on the applicability of drug delivery systems in treating and preventing bacterial, fungal, viral, and parasitic infections, and updating the literature with the newest developments in the field.

## 2. Nanoparticles for Antimicrobial Applications

NPs represent a key component in developing innovative anti-infectious strategies merging therapeutics and new materials toward enhancing antimicrobial potential. Their advantageous intrinsic properties, such as the high specific surface area in relation to volume and increased particle surface energy, render these materials more reactive and effective than their bulk counterparts [37,38,39]. Moreover, their small size is very suitable for antimicrobial biological operations, allowing NPs to interact with biological systems at the molecular level, permitting the targeted delivery of drugs and genes, and ensuring passage through biological barriers [39,40,41,42,43,44]. 

**Figure 1:**
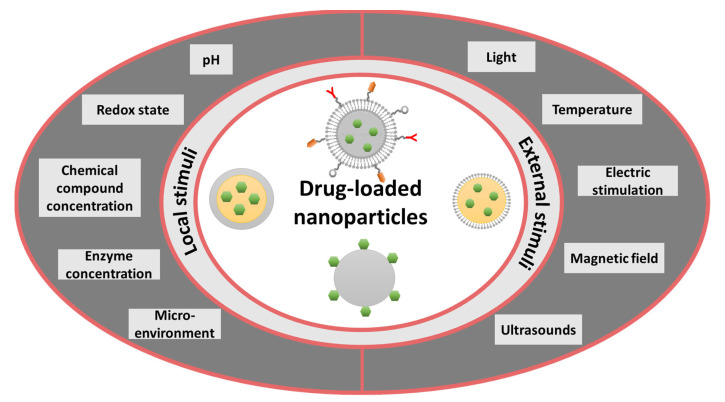
The main types of stimuli-responsive nanoparticles (NPs) for drug delivery.

Moreover, the efficacy of NPs as antimicrobial agents’ carriers can be improved for specific goals (e.g., increased cellular uptake, selective recognition, non-cytotoxicity, better payload binding capacity) through various surface-functionalization approaches. Specifically, stimuli-responsive ligands or functional groups can be used for modifying the surface layer of NPs through different physical, chemical, or biological methods toward achieving optimal antimicrobial activity [31]. In this respect, two main stimulation approaches have been actively researched for delivery nanosystems: locally stimulated or externally stimulated (Figure 1). The first category assumes cargo release in response to chemical and biochemical stimuli at intracellular (e.g., enzymatic activities, hydrolysis, pH, etc.) or tissue level (i.e., specific microenvironmental changes associated with pathological conditions). In contrast, the second category of delivery vehicles supposes the activated targeting and sustained release under the influence of external stimuli, including magnetic fields, electric stimulation, ultrasound, light, and temperature [11,45]. 

**Figure 2:**
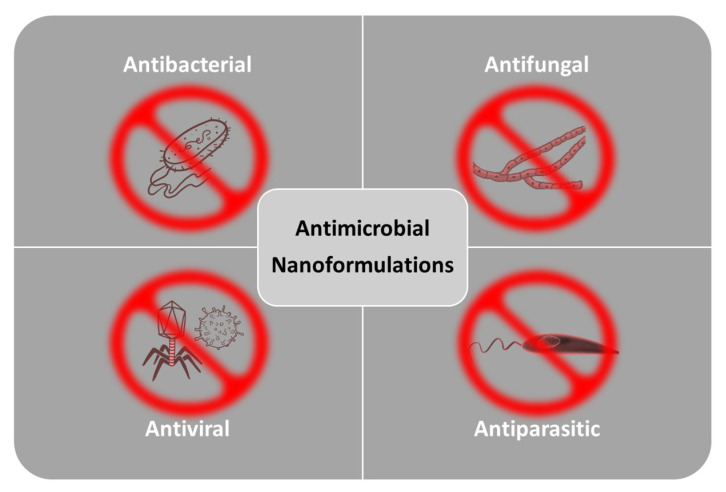
Antimicrobial nanoformulation possibilities.

Taking into account the inherent beneficial properties of NPs and the variety of surface engineering possibilities, numerous studies have developed a wide range of antimicrobial nanoformulations aiming to bring effective solutions against relevant infections (Figure 2). 

In this respect, the following subsections emphasize the recent progress in the fabrication of nanosystems for the delivery of antibacterial, antifungal, antiviral, and antiparasitic agents.

### 2.1. Brief Overview of Nanoparticles Synthesis Methods 

When discussing nanomaterials fabrication, two main approaches can be distinguished: top-down and bottom-up. The top-down approach implies starting from larger structures and reducing their size by means of mechanical force and the aid of finer and finer tools until reaching dimensions in the nano range [46,47]. These methods are preferred in industrial settings, as they can be easily scaled-up and produce fine particles with fine particle-producing capacity and reproducibility. Nonetheless, expensive equipment and intensive energy are required in such processes without guaranteeing control over particle growth and products’ purity [46,48]. In opposition to top-down techniques, bottom-up processes assume the fabrication of nanoparticles through the growth and self-assembly of smaller components of atomic or molecular dimensions, conforming to a natural physical principle or an externally applied driving force [47]. Such methods are simple, rapid, energy-efficient, and cost-effective, being ideal options for laboratory-scale production of amorphous particles with reduced dimensions, narrow particle size distribution, increased solubility, and enhanced bioavailability. However, NPs obtained in this manner tend to agglomerate and might also present stability issues, while there are also several drawbacks associated with the fabrication processes (e.g., low yield, interbatch variability, scaling up challenges) [46,48].

**Figure 3:**
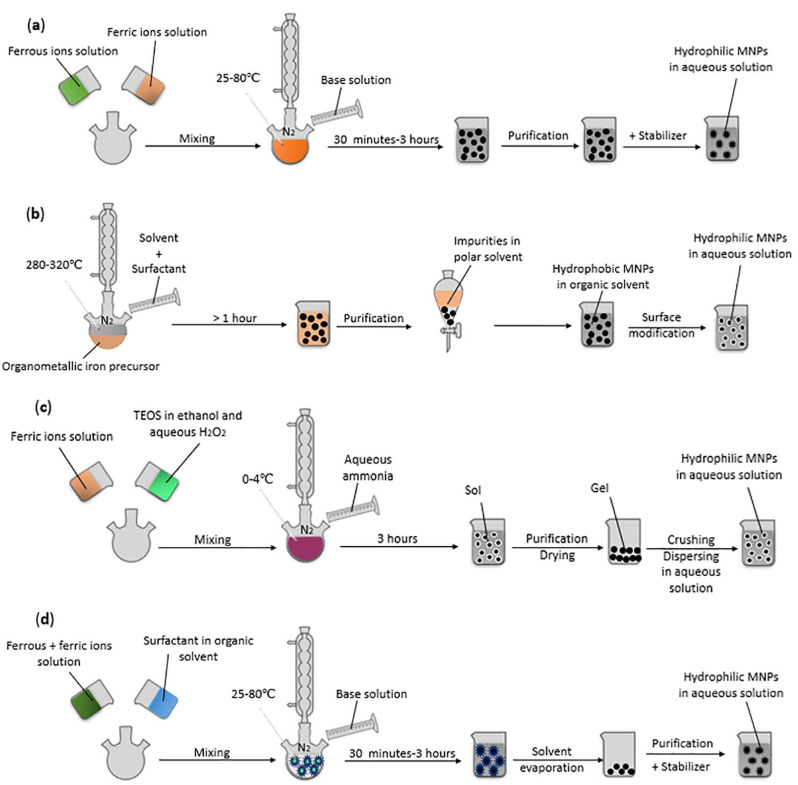
Schematic representation of commonly used synthesis methods to produce magnetite nanoparticles (MNPs): (**a**) co-precipitation; (**b**) thermal decomposition; (**c**) sol–gel; (**d**) microemulsion. Reprinted with permission from [49], © Elsevier, 2022.

**Figure 4:**
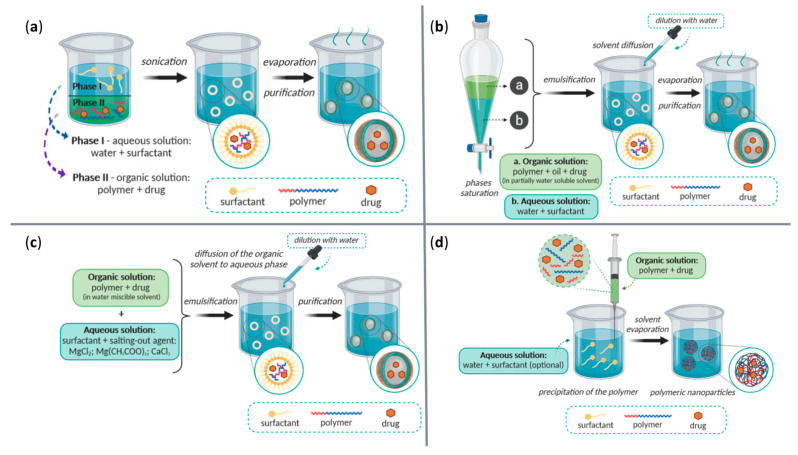
Schematic representation of commonly used synthesis methods to produce polymeric NPs: (**a**) solvent evaporation method; (**b**) emulsification/reverse salting-out method; (**c**) emulsification/solvent diffusion method; (**d**) nanoprecipitation method. Reprinted from an open-access source [50], adapted from [51].

**Figure 5:**
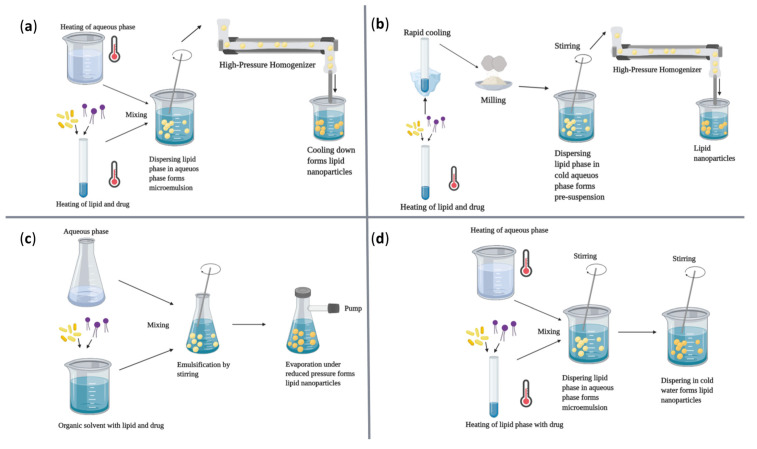
Schematic representation of commonly used synthesis methods to produce lipid-based NPs: (**a**) hot high-pressure homogenization method; (**b**) cold high-pressure homogenization method; (**c**) solvent evaporation method; (**d**) microemulsion method. Reprinted from an open-access source [52], adapted from [53].

A variety of physical, chemical, and biological fabrication methods are available for the synthesis of nanostructures (Table 1), including co-precipitation, hydrothermal synthesis, inert gas condensation, sputtering, microemulsion, microwave-assisted, laser ablation, sol-gel, ultrasound, spark discharge, template synthesis, and biological synthesis [39,46]. Depending on the chemical nature of the NPs, desired properties of the final product, and the cost-effectiveness of fabrication steps, one may prefer one method over the others. Some of the most employed techniques for the fabrication of magnetic NPs, polymeric NPs, and lipid-based NPs, have been gathered in Figure 3, Figure 4 and Figure 5, respectively, offering a visual perspective on nanoconstructs’ synthesis.

Moreover, for producing NPs with tailored structures, mixed approaches can be employed, generally involving a preprocessing step followed by a high-energy step. As each method has its advantages and limitations, thoughtful consideration is required when choosing the synthesis method so that the final nanostructures would have physicochemical stability, low polydispersity, reproducible size, high purity, and optimum morphology for antimicrobial drug delivery purposes [48].

### 2.2. Antibacterial Nanoformulations

Bacterial infections represent one of the biggest global health problems, remaining a significant cause of morbidity and mortality despite the numerous available antibiotics [54]. This is primarily due to the appearance of multi-drug resistant bacterial strains that cannot be effectively treated with conventional therapeutics [40,55,56,57]. Improper prescription of antimicrobial drugs and overuse and/or misuse of antibiotics has led to the current antimicrobial resistance growing crisis, enhancing microbial virulence and allowing bacteria to evade the host’s immune response under the protection of a biofilm [40,56,58,59,60,61]. 

Therefore, a different approach had to be taken to enhance the antibacterial properties of existing drugs. Nanomaterials have become an attractive solution for transporting and releasing hydrophilic and lipophilic antibiotics or natural antimicrobial agents, as they can overpower bacterial resistance through several mechanisms. In more detail, NPs can ensure targeted delivery, allow passage through biological barriers, permeate and destroy the bacterial cell membrane, induce antimicrobial effects within cells, and impede biofilm formation [8,41,62,63,64,65,66,67].

Inorganic nanomaterials such as metal and metal oxide NPs have been of particular interest in creating antibacterial nanoformulations given their advantageous properties (e.g., low cost, long duration, safety, intrinsic antimicrobial activity) [59,68]. These materials act upon bacterial cells mainly through metal ions release, further increasing reactive oxygen species production and affecting bacterial metabolism. Nonetheless, repeated exposure can cause developing resistance even against these NPs. Thus, their antimicrobial efficacy is often enhanced through surface functionalization [69].

Some of the most commonly employed inorganic NPs for antibacterial applications are based on silver, iron oxide, zinc oxide, titanium oxide, magnesium oxide, and silica [69,70,71]. Drugs can be loaded into these systems either as coatings/shells on the NP surface [72], or they can be incorporated into the pores of the material [73]. Various synthetic antimicrobial agents have been used as cargos, including streptomycin [72], neomycin [72], vancomycin [74], cephalexin [75], and ciprofloxacin [76], leading to stronger effects against numerous pathogens. Specifically, antibiotic-loaded inorganic NPs have been tested with promising results against relevant bacterial strains, counting *Staphylococcus aureus* (*S. aureus*) [72,73,75,76], *Pseudomonas aeruginosa* (*P. aeruginosa*) [72], *Bacillus subtilis* (*B. subtilis*) [74], *Bacillus cereus* (*B. cereus*) [75], *Streptococcus* [74], *Escherichia coli* (*E. coli*) [73,74,75], and *Salmonella typhimurium* (*S. typhimurium*) [75]. 

Moreover, the nanosystems’ antimicrobial properties and safety can be enhanced by adding biocompatible coatings [72,75,77]. In addition, surface functionalization of NPs can be performed to bypass triggering host defense mechanisms until reaching the site of infection and avoid potential adverse reactions or inhibition of NPs bioactivity [66,78,79,80].

Polymers represent another class of highly convenient materials for fabricating antimicrobial drug-delivery NPs. The main advantages of polymeric materials reside in their variety, versatility, and ease of functionalization. These characteristics render polymers suitable for improving drug solubility, delivering the cargo to the desired site, and targeting bacterial pathogens [50,67,81]. 

Recent studies have focused on developing vehicles from natural polymers, as they are generally recognized to possess superior biocompatibility to synthetic materials. The most commonly employed natural polymers for antibacterial agents encapsulation are polysaccharides, chitosan and alginate being the choice of numerous studies [55,82,83,84,85,86,87,88,89,90,91,92,93]. Nonetheless, synthetic polymers such as polylactic acid (PLA) [94,95], poly(lactic-co-glycolic acid (PLGA) [96,97], and polyvinylpyrrolidone (PVP) [98] have also attracted research interest as nanocarriers. 

In what concerns the freight, a broad range of antimicrobial agents has been reported in the literature as suitable for polymer encapsulation. Synthetic drugs (e.g., levofloxacin [82], gentamicin [83,92], N′-((5-nitrofuran-2-yl)methylen)-2-benzhydrazide [86], rifampicin [55,94], ascorbic acid [55], doxycycline [88], rifaximin [89], ampicillin [91], teicoplanin [98], camptothecin [99], and vancomycin [100]) and natural antimicrobials (e.g., oregano oil [90,101], *Cinnamomum zeylanicum* (*C. zeylanicum*) essential oil [84], nettle essential oil [85], *Pistacia lentiscus* (*P. lentiscus*) L. var. *chia* essential oil [95], and red propolis extract [96]) have been successfully incorporated into nanosized polymeric materials. 

The synergic properties of engineered polymeric NPs and carried antibacterial moieties have led to the obtaining of promising candidates for anti-infective therapeutics against *S. aureus* [55,82,83,85,86,87,90,91,92,94,96,97,99,100], *E. coli* [83,84,85,89,90,92,95,99], *P. aeruginosa* [82,89,90,92,93,96], *Erwinia carotovora* (*E. carotovora*) [84], *Pseudomonas fluorescens* (*P. fluorescens*) [84], *Enterococcus faecalis* (*E. faecalis*) [88,90], *Proteus mirabilis* (*P. mirabilis*) [88], *Bacillus haynesii* (*B. haynesii*) [89], *Streptococcus pyogenes* (*S. pyogenes*) [90], *Yersinia enterocolitica* (*Y. enerocolitica*) [90], *Listeria monocytogenes* (*L. monocytogenes*) [92], *B. subtilis* [95], *Streptococcus pneumoniae* (*S. pneumoniae*) [98], *Haemophilus influenzae* (*H. influenzae*) [98], and *Klebsiella pneumonia* (*K. pneumoniae)* [99]. 

**Figure 6:**
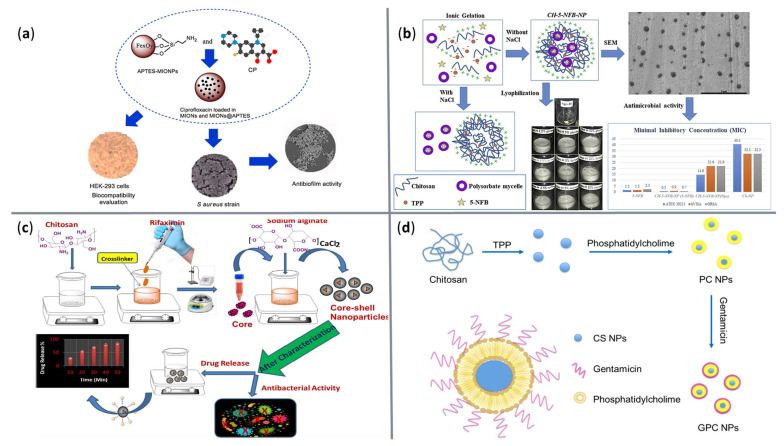
Visual representation of several nanosystems for antibacterial agents’ delivery. (**a**) Mesoporous iron oxide NPs loaded with ciprofloxacin. Reprinted with permission from [76], © Elsevier, 2021. (**b**) Polysorbate 20 micelles loaded in chitosan NPs for N′-((5-nitrofuran-2-yl)methylen)-2-benzhydrazide delivery. Reprinted with permission from [86], © Elsevier, 2020. (**c**) Chitosan-alginate core-shell NPs loaded with rifaximin. Reprinted with permission from [89], © Elsevier, 2021. (**d**) Phosphatidylcholine-chitosan liposome NPs for gentamicin delivery. Reprinted with permission from [92], © Elsevier, 2020.

To emphasize the variety and versatility of recently developed antibacterial nanoformulations, Table 2 summarizes several studies that fabricated promising drug delivery nanosystems for fighting against clinically relevant strains. In addition, Figure 6 provides a visual perspective over some of the discussed nanoconstructs. 

### 2.3. Antifungal Nanoformulations

Fungal infections represent a significant health issue, being associated with high morbidity and mortality. Immunocompromised hosts are particularly susceptible to invasive infections, with the mortality rates in such patients going above 60% in certain situations [3,102,103]. The conventional approach in treating such infections assumes the administration of antifungal agents such as polyenes, azoles, and echinocandins [104]. Nevertheless, these drugs present a series of disadvantages that impede their therapeutic action. Conventional antifungals exhibit non-neglectable toxicity, adverse side effects, acquired resistance, and unclear effects in immunocompromised patients [105,106,107,108]. 

Therefore, recent research tried to solve these issues by orienting to safer strategies, including encapsulation into biocompatible NPs and replacement with natural alternatives. In this regard, scientists have explored a plethora of nanomaterials for designing performant delivery systems for antifungal agents. Promising results have been reported when using metal and metal oxide-based NPs [109,110], natural polymers [90,111,112], biocompatible synthetic polymers [113,114,115,116,117,118], and lipid-based nanocarriers [119,120]. Regarding the choice of antimicrobial agents, most studies elaborated nanoconstructs for the delivery of synthetic drugs (e.g., nystatin [109], fluconazole [109], amphotericin B [110,117], voriconazole [111], itraconazole [115,116], ketoconazole [118], miconazole nitrate [119], clotrimazole [112]), but several natural antifungal agents (e.g., seedless *Vitis vinifera* (*V. vinifera*) [112], oregano oil [90], *Lippia sidoides* (*L. sidoides*) essential oil [120], pterostilbene [113], farnesol [114]) have also been investigated and led to promising results.

In what concerns the species of interest, most studies have focused on developing antifungal nanoformulations targeting *Candida albicans* (*C. albicans*) [90,109,110,111,112,114,115,117,118,119], as candidiasis is among the most common invasive mycotic diseases, and *C. albicans* is recognized as the leading cause of invasive candidiasis [121,122]. Nonetheless, other fungal pathogens have also been considered, including *Aspergillus brasiliensis* (*A. brasiliens*) [109,113], *Aspergilus niger* (*A. niger*) [112], *Cryptococcus neoformans* (*C. neoformans*) [110], *Histoplasma capsulatum* (*H. capsulatum*) [116], *Trichophyton rubrum* (*T. rubrum*) [118], *Trichophyton mentagrophytes* (*T. mentagrophytes*) [118], *Microsporum gypseum* (*M. gypseum*) [118], *Candida dubliniensis* (*C. dubliniensis*) [118], *Candida krusei* (*C. krusei*) [118], *Candida parapsilosis* (*C. parapsilosis)* [118], *Candida tropicalis* (*C. tropicalis)* [118], and *Candida auris* (*C. auris*) [120].

**Figure 7:**
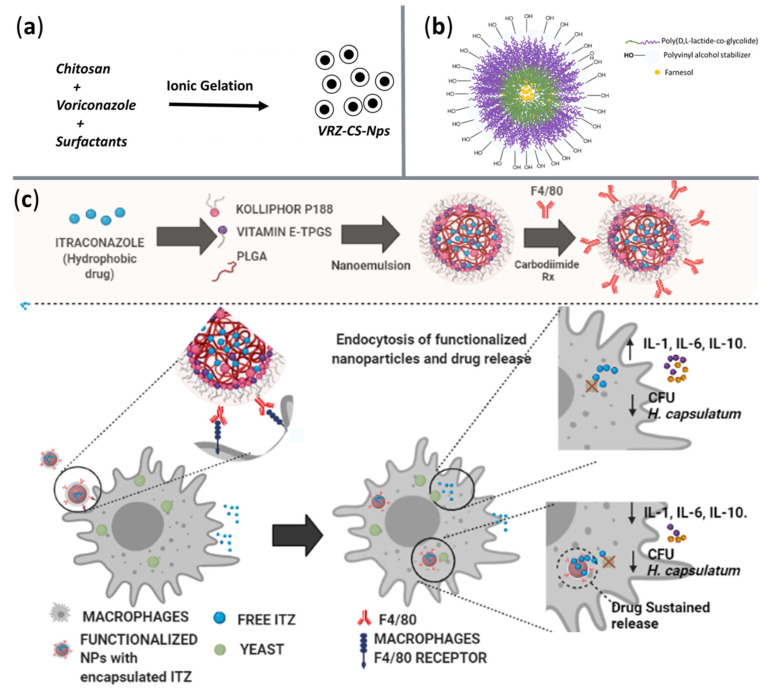
Visual representation of several nanosystems for antifungal agents’ delivery. (**a**) Chitosan-based NPs loaded with voriconazole. Adapted from an open-access source [111]. (**b**) PLGA NPs loaded with farnesol. Reprinted from an open-access source [114]. (**c**) PLGA NPs functionalized with anti-F4/80 antibodies loaded for itraconazole delivery. Reprinted from an open-access source [116].

For clarity, Table 3 correlates NP material, physicochemical properties of the delivery nanosystems, carried antifungal agents, and targeted pathogens, while Figure 7 schematically illustrates a few of these nanostructures. 

### 2.4. Antiviral Nanoformulations

Viruses are another class of dangerous pathogens, as they are responsible for around two million deaths per year [123]. Their small size allows viruses to enter the human body through various routes and internalize into living cells. Some of the most pathogenic viruses include human immunodeficiency virus (HIV), human papillomavirus (HPV), herpes simplex virus (HSV), norovirus, hepatitis viruses, and coronaviruses, leading to significant morbidity and mortality. Moreover, the occurrence of viral outbreaks was seen to have devastating effects from economic and social points of view [3,124]. 

In the fight against infections, NPs offer certain advantages for delivering antivirals to the target sites as they have the ability to surpass biological barriers thanks to their small size and tailored surface characteristics. Through their unique properties, NPs allow antivirals to be released at the infection site, followed by their attachment to viral receptors on the surface of host cells or internalization within the cell resulting in the disruption of the viral replication cycle [124]. 

Taking into account the benefits of NPs, several research studies explored the antimicrobial potential of a number of antiviral drugs encapsulated in different nanomaterials. Examples of investigated nanocarriers include silver NPs [125], titanium dioxide NPs [126], oligo- and polysaccharide-based NPs [127,128,129,130,131], solid lipid NPs (SLNs) [132,133], and large unilamellar vesicles [134]. The ingenious association with antivirals (e.g., docetaxel [125], flavonoids [126], zidovudine [127,128], dolutegravir sodium [129], efavirenz [130], acyclovir [131,132], ritonavir [133]) and functionalization agents has led to the obtaining of promising anti-infective therapeutic nanoformulations. Specifically, the proposed delivery systems targeted clinically relevant viruses, such as severe acute respiratory syndrome coronavirus 2 (SARS-CoV-2) [125,126], HIV [127,128,129,130,133,134,135], and HSV [131,132].

**Figure 8:**
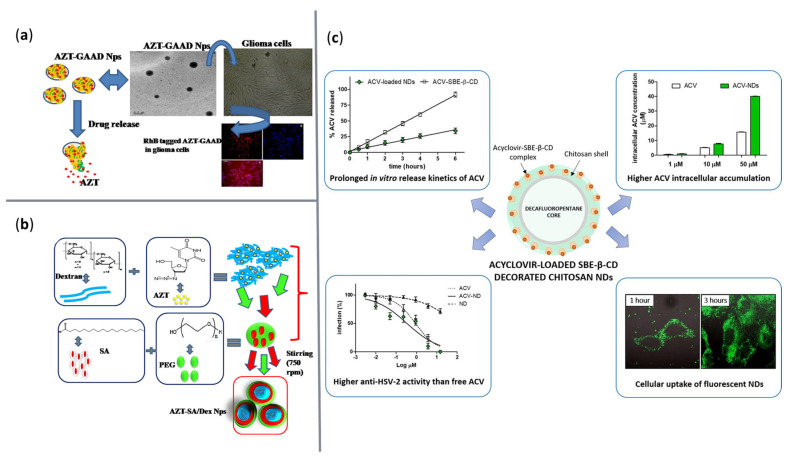
Visual representation of several nanosystems for antiviral agents’ delivery. (**a**) Amide-functionalized alginate NPs loaded with zidovudine. Reprinted with permission from [127], © Elsevier, 2018. (**b**) Dextran-stearic acid core shell NPs loaded with zidovudine. Reprinted with permission from [128], © Elsevier, 2018. (**c**) Sulfobutyl ether-β-cyclodextrin decorated chitosan nanodroplets for acyclovir delivery. Reprinted with permission from [131], © Elsevier, 2020.

To better clarify the features of the newly developed antiviral drug delivery systems, Table 4 and Figure 8 summarize several examples of studies in the field. 

### 2.5. Antiparasitic Nanoformulations

Aside from bacteria, fungi, and viruses, several parasites have also been recognized for their infective potential. One example is represented by *Leishmania* spp., which comprises a group of flagellated protozoans responsible for neglected tropical diseases known as leishmaniasis. Characterized by high mortality, disability, and morbidity rates, leishmaniasis represents a major global health concern, being endemic in 102 countries worldwide [136,137,138]. Consequently, scientific interest arose in finding antiparasitic solutions able to effectively and efficiently fight against *Leishmania*. 

For instance, Badirzadeh et al. [137] proposed coating silver NPs with curcumin. In vitro and in vivo tests performed on mouse models demonstrated encouraging results, the nanoformulation significantly reducing the burden of promastigotes and amastigotes of *Leishmania* parasite in a single treatment. Alternatively, Snoussi et al. [136] prepared silver-loaded biochar that exhibited strong antiparasitic activity against the promastigotes stage of *Leishmania donovani*, *Leishmania amazonensis,* and epimastigotes of *Trypanosoma cruzi*. On a different note, Durak and colleagues [139] encapsulated two active ingredients with antibacterial and antiparasitic activities (i.e., caffeic acid phenethyl ester and juglone) into single polymeric NPs. These multifunctional nanoformulations proved synergistic activity, being promising candidates for antiparasitic therapy. 

**Figure 9:**
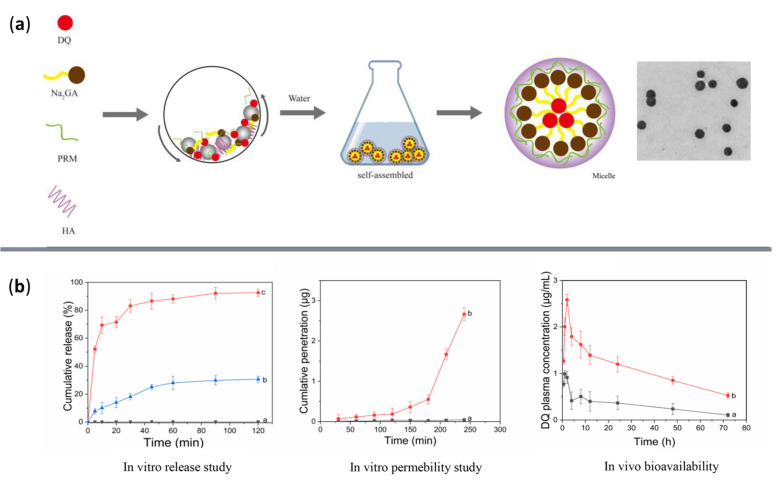
(**a**) Visual representation for the fabrication of decoquinate delivery system and (**b**) systematic testing. Reprinted with permission from [142], © Elsevier, 2022.

Several studies have also directed their efforts toward creating delivery nanosystems aimed at other pathogens. For example, Kanwal et al. [140] reported the fabrication of silver NPs conjugated with novel bisindole and thiazole derivatives as potential antiamoebic formulations with enhanced activity against *Balamuthia mandrillaris* and *Naegleria fowleri*. In contrast, Real et al. [141] developed a drug delivery system for treating fascioliasis. For this purpose, the researchers loaded triclabendazole into nanocapsules, enhancing its bioavailability and lowering its cytotoxic effects compared to the free drug. Differently, Wei et al. [142] created a nanocarrier for decoquinate, a drug known to have control effects on hematogeneous parasites. The authors encapsulated the antiparasitic agent into disodium glycyrrhizinate NPs with protamine and anionic hyaluronic acid layers (Figure 9), significantly increasing the drug’s bioavailability, ensuring a higher concentration in the blood and preferential liver tissue accumulation. 

## 3. Discussion

Humans are exposed to numerous pathogens that can trigger burdensome bacterial, fungal, viral, and parasitic infections. Conventional treatment approaches revolve around the systemic administration of synthetic drugs that, due to the emergence of drug-resistant microbial strains, exhibit low efficacy, in addition to the disadvantages of poor solubility, toxicity, and adverse effects. In this context, nanotechnology started being increasingly explored for designing improved antimicrobial agents

**Figure 10:**
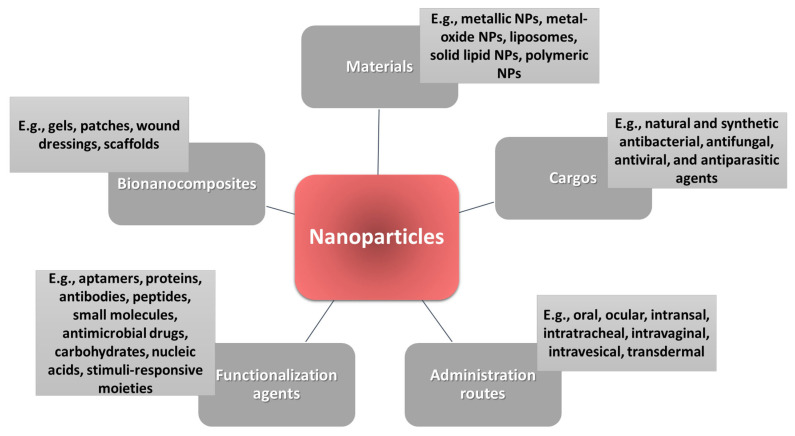
Overview of the possibilities of using NPs as delivery vehicles for antimicrobial applications.

NPs of many sorts (Figure 10) have been recently developed as performant carriers of numerous antimicrobial agents, holding promise for improved strategies to combat a wide range of infectious diseases. Nanodimensional materials, such as metallic NPs, metal-oxide NPs, lipid-based NPs, and polymeric NPs, have attracted considerable interest in recent years for fabricating delivery vehicles. Specifically, the variety and versatility of nanomaterials have been extensively explored by researchers for creating innovative therapeutic formulations that can be administrated on different routes, including oral [2,141,142], ocular [82,83,143], intranasal [125,129,130], intratracheal [55,144], intravaginal [145,146], intravesical [147,148], and transdermal [149,150] routes. Compared to free drugs, NP-loaded antimicrobial agents can be administered in so many ways due to their increased safety, reduced systemic adverse effects, enhanced solubility, and improved bioavailability. Moreover, the various natural and synthetic antimicrobial cargos can be released in a targeted manner by adding various functionalization agents onto the nanocarriers’ surface. Functionalization agents can also work in synergy with the core delivery system, increasing therapeutic efficacy and reducing drug resistance [11,31]. 

In addition to their stand-alone utility, NPs may further be incorporated in different other materials to create bionanocomposites with enhanced antimicrobial properties. In this respect, researchers propose the use of various nanostructured gels [82,83,98,100,151], patches [149,150,152], wound dressings [153,154,155,156], and scaffolds [157,158,159] as alternative solutions for treating and preventing microbial infections. A particularly exploited application of NPs is the fabrication of coatings for creating surfaces with antimicrobial and antibiofilm properties [12,19,27,29,77,160,161,162,163,164]. Even though aimed mainly at the modification of biomedical devices, such as catheters, implants, and prostheses, applying antimicrobial coatings can also be of high utility in covering other contact surfaces. For instance, they can be used to prevent pathogens from spreading from day-to-day objects, including doorknobs, packaging, and handrails [37]. 

## 4. Conclusions and Future Perspectives

To summarize, various NPs have been investigated as drug delivery vehicles to surpass traditional drugs’ limitations. Numerous studies have successfully loaded natural and synthetic drugs into inorganic, lipid, and polymeric-based nanosystems, obtaining promising results against a broad range of pathogens, but mostly bacterial strains. 

To conclude, there is an increased research interest in developing alternative antimicrobial agents, and current progress demonstrates the great potential of nanostructured materials in preventing and treating infectious diseases. Nonetheless, there is still room for improvement in the field, especially concerning the expansion of antiviral, antifungal, and antiparasitic applications of drug delivery nanosystems. Further studies should also focus on managing complex and mixed biofilms, an understudied and challenging niche of microbial infections. Moreover, being so new, most of the discussed nano-therapeutic options have not yet advanced beyond preclinical testing. Thus, rigorous additional studies are required before they become clinically and commercially available solutions. 

## Figures and Tables

**Table 1:** Classification of NP synthesis methods.

Synthesis Approach	Nature of Involved Processes	Examples of Techniques
Top-down approach	Physical methods	Ball milling, Laser ablation, Electron beam deposition, Sputtering, Aerosol spray
Bottom-up approach	Chemical methods	Co-precipitation, Thermal decomposition, Sol-gel, Microemulsion Sonochemical, Hydrothermal, Microwave assisted, Chemical reduction, Electrochemical, Solvothermal
	Biological methods	Bacteria-based, Plant-based

**Table 2:** Examples of antibacterial nanoformulations.

NP Type	Fabrication Method	Physicochemical Properties	Antimicrobial Agent(s)	Targeted Pathogen(s)	Ref.
Magnetite NPs	Co-precipitation	Size range: ~2.8–~4.7 nm Shape: spherical	Streptomycin/ neomycin	*S. aureus, P. aeruginosa*	[72]
Magnetite NPs	Co-precipitation	Size range: 10–20 nm Average hydrodynamic diameter: 39.3 nm	Vancomycin	*B. subtilis, Streptococcus, E. coli*	[74]
Basil seed mucilage coated magnetite NPs	Co-precipitation	Mean size: 6 nm Specific surface area: 30.60 m^2^g^−1^	Cephalexin	*E. coli, S. typhimurium, S. aureus, B. cereus*	[75]
Mesoporous iron oxide NPs	Co-precipitation	Average size: 78.34 ± 1.38 nm Zeta potential: −18.45 ± 1.89 mV Superficial area: 258.27 ± 7.51 m^2^g^−1^	Ciprofloxacin	*S. aureus*	[76]
Silanized mesoporous iron oxide NPs	Co-precipitation	Average size: 86.32 ± 2.0 nm Zeta potential: +5.76 ± 0.65 mV Superficial area: 186.27 ± 6.68 m^2^g^−1^	Ciprofloxacin	*S. aureus*	[76]
CTAB-loaded mesoporous silica NPs	Hydrothermal method	Size range: ~100–110 nm Shape: quasi-spherical	Silver NPs	*E. coli, S. aureus*	[73]
Chitosan NPs	Ionic gelation	Mean size: ranging from 161.90 ± 3.32 nm to 283.97 ± 4.21 nm Zeta potential: ranging from +30.43 ± 1.08 to +21.87 ± 1.87 mV	Levofloxacin	*P. aeruginosa, S. aureus*	[82]
Chitosan NPs	Ionotropic gelation	Average size: 135.2 ± 3.24 nm Shape: spherical	Gentamicin	*E. coli, S. aureus*	[83]
Chitosan NPs	Ionic gelation	Size range: 20–80 nm Hydrodynamic diameter: 141.4–181.6 nm Zeta potential: ranging from +49.9 to +38.7 mV Shape: spherical	*C. zeylanicum* essential oil	*E. coli, E. carotovora, P. fluorescens*	[84]
Chitosan NPs	Two-stage emulsion-ionic gelation method	Mean size: ranging from 208.3 ± 44.5 to 369.4 ± 48.1 nm Zeta potential: ranging from +30.1 ± 2.3 to +14.46 ± 0.9 mV	Nettle essential oil	*E. coli, S. aureus*	[85]
Polysorbate 20 micelles loaded in chitosan NPs	Ionic gelation	Average size: 321 nm Zeta potential: +37 mV Shape: spherical	N′-((5-nitrofuran-2-yl)methylen)-2-benzhydrazide	Multidrug-resistant *S. aureus*	[86]
Alginate-chitosan NPs	Ionic gelation	Average hydrodynamic diameter: 380 ± 15 nm Zeta potential: −28.5 ± 0.03	Rifampicin and ascorbic acid	MSSA, MRSA	[55]
Alginate-chitosan NPs	Calcium ion-induced pre-gelation of alginate core and further complexation with chitosan	Average hydrodynamic diameter: 276.5 ± 42 nm Zeta potential: −25 mV	LysMR-5	*S. aureus*	[87]
Chitosan-alginate NPs	Ionotropic gelation	Average size: 61.9 nm	Doxycycline	*E. faecalis, P. mirabilis*	[88]
Chitosan-alginate core-shell NPs	Precipitation/coacervation method	Size range: 700–1150 nm Zeta potential: −16.61 mV	Rifaximin	*E. coli, P. aeruginosa, Bacillus haynesii*	[89]
Chitosan-alginate NPs	Emulsification and consequent electrostatic gelation	Average size: 320 nm Zeta potential: −25 mV	Oregano oil	MSSA, MRSA, *E. faecalis, S. pyogenes, E. coli, P. aeruginosa, Y. enterocolitica*	[90]
Chitosan-polyanion NPs	Ionic gelation and polyelectrolyte complexation assisted by high-intensity sonication	Average size: ranging from 130.7 to 249.2 nm Zeta potential: ranging from +39.5 to +49.2 mV	Ampicillin	*S. aureus*	[91]
Phosphatidylcholine-chitosan liposome NPs	Ionic gelation	Average size: ~140 nm Zeta potential: −19.5 mV	Gentamicin	*L. monocytogenes, S. aureus, P. aeruginosa, E. coli*	[92]
Dextran NPs	Ionic gelation	Average size: 18 nm Zeta potential: −13 mV	SET-M33 peptide	*P. aeruginosa*	[93]
PLA NPs functionalized with poly-L-lysine	Surfactant-free nanoprecipitation	Average hydrodynamic diameter: 162 ± 2 nm Zeta potential: +40 ± 2 mV	Rifampicin	*S. aureus*	[94]
PLA/PVA NPs	Solvent evaporation method	Average size: 239.9 nm Zeta potential: −29.1 mV	*P. lentiscus* L. var. *chia* essential oil	*E. coli, B. subtilis*	[95]
PLA/lecithin	Solvent evaporation method	Average size: 286.1 nm Zeta potential: −34.5 mV	*Pistacia lentiscus* L. var. *chia* essential oil	*E. coli, B. subtilis*	[95]
PLGA NPs	Emulsification solvent diffusion method	Average size: 69.2 nm Average hydrodynamic diameter: 224.23 ± 18.87 nm Zeta potential: −32.1 ± 4.53 mV Shape: spherical	Red propolis extract	*S. aureus, P. aeruginosa*	[96]
PLGA NPs functionalized with specific aptamers	Oil-in-water emulsification-evaporation method	Average hydrodynamic size: 226.00 ± 5.57 nm Zeta potential: 29.00 ± 1.18 mV	Teicoplanin	*S. aureus*	[97]
PVP-coated silver NPs	Chemical reduction	Average size: 9.23 ± 2.03 nm Shape: spherical	Silver NPs	*S. pneumoniae, H. influenzae*	[98]

Abbreviations: CTAB—cetyltrimethylammonium bromide; MRSA—methicillin-resistant *Staphylococcus aureus*; MSSA—methicillin-sensitive *Staphylococcus aureus*; NP—nanoparticle; PLA—polylactic acid; PLGA—poly(lactic-co-glycolic acid); PVA—poly(vinyl alcohol); PVP—polyvinylpyrrolidone.

**Table 3:** Examples of antifungal nanoformulations.

NP Type	Fabrication Method	Physicochemical Properties	Antimicrobial Agent(s)	Targeted Pathogen(s)	Ref.
Silver NPs	Chemical reduction	Average size: 80 nm Shape: spherical	Nystatin	*C. albicans, A. brasiliensis*	[109]
Silver NPs	Chemical reduction	Average size: 25 nm Shape: spherical	Fluconazole	*C. albicans, A. brasiliensis*	[109]
ZnO-PEGylated NPs	Nanoemulsification	Average size: 662.3 ± 24.7 nm Zeta potential: −14.2 ± 0.94 mV	Amphotericin B	*C. albicans, C. neoformans*	[110]
Chitosan-based NPs	Ionic gelation	Average size: ranging from 167 ± 8.23 to 475 ± 15.30 nm Zeta potential: ranging from 39 ± 2.56 to 45 ± 3.11 mV Shape: spherical	Voriconazole	*C albicans*	[111]
Chitosan NPs	Ionic gelation	Average size: 35.4 nm Zeta potential: +31 mV	Seedless *V. vinifera* and clotrimazole	*C. albicans, A. niger*	[112]
Chitosan-alginate NPs	Emulsification and consequent electrostatic gelation	Average size: 320 nm Zeta potential: −25 mV	Oregano oil	*C albicans*	[90]
PLGA NPs	n/r	Average size: 50 nm Zeta potential: −25 mV	Coumarin 6 and pterostilbene	*A. brasiliensis*	[113]
PLGA NPs	Emulsion evaporation method	Average size: 140 nm	Farnesol	*C albicans*	[114]
PLGA NPs	Nanoprecipitation and single emulsion solvent evaporation methods	Average size: 176.96 ± 24.32 nm Zeta potential: −24.7 ± 1.04 mV Shape: spherical	Itraconazole	*C albicans*	[115]
PLGA NPs functionalized with anti-F4/80 antibodies	Nanoemulsion	Average size: 226.66 ± 13.05 nm Zeta potential: −27.9 ± 0.26 mV	Itraconazole	*H. capsulatum*	[116]
Aptamer-functionalized PLGA-PEG NPs	Double emulsification method	Average size: 273.9 ± 1.14 nm Zeta potential: −20 mV	Amphotericin B	*C albicans*	[117]
PLA NPs	Nanoprecipitation	Mean size: 188.5 nm Zeta potential: 4.80 mV Shape: spherical	Ketoconazole	*T. rubrum, T. mentagrophytes, M. gypseum, C. albicans, C. dubliniensis, C. krusei, C. parapsilosis, C. tropicalis*	[118]
SLNs	High shear homogenization and ultrasonication	Average size: ranging from 244.2 ± 27.2 to 493.6 ± 35.3 nm Zeta potential: ranging from −21.6 ± 7.05 to −1.4 ± 6.84 mV	Miconazole nitrate	*C. albicans*	[119]
NLC	Hot emulsification method	Average size: ranging from 213.1 ± 1.7 to 445.5 ± 8.7 nm Zeta potential: ranging from −63.8 ± 8.7 to −93.1 ± 2.7 mV	*L. sidoides* essential oil	*C. auris*	[120]

Abbreviations: n/r—not reported; NLC—nanostructured lipid carriers; NP—nanoparticle; PEG—polyethylene glycol; PLA—polylactic acid; PLGA—poly(lactic-co-glycolic acid); SLN—solid lipid nanoparticle.

**Table 4:** Examples of antiviral nanoformulations.

NP Type	Fabrication Method	Physicochemical Properties	Antimicrobial Agent(s)	Targeted Pathogen(s)	Ref.
NH_2_-functionalized silver NPs	UV irradiation and chemical reduction	Average hydrodynamic diameter: 5.0 nm Zeta potential: 22 mV	Docetaxel	SARS-CoV-2	[125]
Amide-functionalized alginate NPs	Emulsion solvent evaporation method	Average size: ranging from 437 ± 2.3 to 473 ± 2.6 nm Zeta potential: ranging from −42.16 ± 3.2 to −34.13 ± 1.61 mV Shape: spherical	Zidovudine	HIV	[127]
Dextran-stearic acid core shell NPs	Double emulsion solvent evaporation method	Average size: ranging from 356 ± 2.06 to 730 ± 1.22 nm Zeta potential: ranging from −31.6 ± 2.12 to −20.9 ± 1.7 mV	Zidovudine	HIV	[128]
HPβCD NPs crosslinked with diphenyl carbonate	Cross-linking	Average size: ranging from 72.47 ± 4.8 to 106.5 ± 5.6 nm Zeta potential: ranging from −30.3 ± 4.1 to −7.77 ± 3.1 mV	Dolutegravir sodium	HIV	[129]
Chitosan-g-HPβCD NPs	Ionic gelation	Average size: ranging from 198 ± 4.4 471.3 ± 5.3 nm Zeta potential: ranging from 3.14 ± 2.6 to 11.5 ± 2.1 mV	Efavirenz	HIV	[130]
Sulfobutyl ether-β-cyclodextrin decorated chitosan nanodroplets	Electrostatic interaction	Average size: 395.4 ± 12.6 nm Zeta potential: 19.98 ± 3.02 mV	Acyclovir	HSV type 2	[131]
SLNs	Emulsification and low-temperature solidification	Average size: 180 ± 1.2 nm Zeta potential: −25 mV	Acyclovir	HSV	[132]
SLNs	Solvent emulsification evaporation and double emulsion methods	Mean size: ranging from 178.7 ± 4.5 to 254.3 ± 16.6 nm Zeta potential: ranging from 39.35 ± 1.2 to 50.80 ± 4.8 mV	Ritonavir	HIV-1	[133]

Abbreviations: HIV—human immunodeficiency virus; HPβCD—2-Hydroxypropyl-beta-cyclodextrin; HSV—herpes simplex virus; LUVs—large unilamellar vesicles; NP—nanoparticle; SARS-CoV-2—severe acute respiratory syndrome coronavirus 2; SLN—solid lipid nanoparticle.

## Data Availability

Not applicable.
